# A Novel System for Measuring Pterygium's Progress Using Deep Learning

**DOI:** 10.3389/fmed.2022.819971

**Published:** 2022-02-14

**Authors:** Cheng Wan, Yiwei Shao, Chenghu Wang, Jiaona Jing, Weihua Yang

**Affiliations:** ^1^College of Electronic Information Engineering, Nanjing University of Aeronautics and Astronautics, Nanjing, China; ^2^Department of Ophthalmology, Nanjing Lishui Hospital of Traditional Chinese Medicine, Nanjing, China; ^3^The Laboratory of Artificial Intelligence and Bigdata in Ophthalmology, The Affiliated Eye Hospital of Nanjing Medical University, Nanjing, China; ^4^Department of Ophthalmology, Children's Hospital of Nanjing Medical University, Nanjing, China

**Keywords:** pterygium, image segmentation, deep learning, chi-square test, computer-aided diagnosis

## Abstract

Pterygium is a common ocular surface disease. When pterygium significantly invades the cornea, it limits eye movement and impairs vision, which requires surgery to remove. It is medically recognized that when the width of the pterygium that invades the cornea is >3 mm, the patient can be treated with surgical resection. Owing to this, this study proposes a system for diagnosing and measuring the pathological progress of pterygium using deep learning methods, which aims to assist doctors in designing pterygium surgical treatment strategies. The proposed system only needs to input the anterior segment images of patients to automatically and efficiently measure the width of the pterygium that invades the cornea, and the patient's pterygium symptom status can be obtained. The system consists of three modules, including cornea segmentation module, pterygium segmentation module, and measurement module. Both segmentation modules use convolutional neural networks. In the pterygium segmentation module, to adapt the diversity of the pterygium's shape and size, an improved U-Net++ model by adding an Attention gate before each up-sampling layer is proposed. The Attention gates extract information related to the target, so that the model can pay more attention to the shape and size of the pterygium. The measurement module realizes the measurement of the width and area of the pterygium that invades the cornea and the classification of pterygium symptom status. In this study, the effectiveness of the proposed system is verified using datasets collected from the ocular surface diseases center at the Affiliated Eye Hospital of Nanjing Medical University. The results obtained show that the Dice coefficient of the cornea segmentation module and the pterygium segmentation module are 0.9620 and 0.9020, respectively. The Kappa consistency coefficient between the final measurement results of the system and the doctor's visual inspection results is 0.918, which proves that the system has practical application significance.

## Introduction

Pterygium is a general ocular surface and degenerative disease characterized by conjunctival fibrovascular proliferation and invasion of the peripheral cornea (1). It is commonly referred to as a proliferatively disorder because of its growth rate and propensity for recurrence (2). Pterygium is composed of a head, neck, and body that invades the cornea, the superficial limbus, and overlies the sclera, respectively (3). The growth of pterygium in the eye can cause foreign body sensation and dryness, limit eye movement, and impair vision (4). When pterygium involves the visual axis, vision is not decreased by the irregular astigmatic refractive aberrations induced on the corneal topography alone, but also by the direct obscuring of the central visual axis (5). The major treatment for pterygium in recent times is surgical resection (1). According to several reports, the incidence of pterygium in some parts of the world has been reported to be as high as 2.1 per 1,000 persons, where up to 0.8 per 1,000 persons have undergone pterygium excision (6). It is widely known in the medical field that when the width of the pterygium (WP) that invades the cornea is >3 mm, the patient can be treated with surgical resection (7).

Therefore, measuring the pathological progress of pterygium is essential for designing surgical treatment strategies for pterygium.

Using computer image processing technology and medical image diagnosis technology, such as three-dimensional modeling and quantitative analyzing, to conduct early diagnosis of various eye diseases can equip doctors with enough information to diagnose patients, formulate effective treatment plans, and make reasonable preparations before surgery. For ocular surface diseases, such as pterygium, clinicians usually take anterior segment images as the basis of auxiliary diagnosis. However, in the absence of professional tools, it becomes difficult for these clinicians to accurately measure the pathological progress of pterygium which is essential for an accurate diagnosis. In recent years, many scholars have studied the characteristics of pterygium and achieved remarkable research success (1–3). However, there are only a few studies on the application of computer image processing technology for analyzing and processing the anterior segment images of pterygium patients. Moreover, most of the contents of these studies focus on the segmentation of the overall pterygium in the anterior segment image (8, 9), and the segmentation of the cornea in the anterior segment image (10, 11); thus, lacking quantitative analysis of the pathological progress of pterygium. In the study of the pterygium segmentation in anterior segment images, most researchers used traditional image segmentation methods. The Dice coefficient of these methods is usually <90%, which means that there is still room for further improvement in the segmentation accuracy. In addition, traditional image segmentation methods require manual design of the relevant model before segmentation occurs, resulting in an increased uncertainty because of the manual operations.

In view of the abovementioned challenges and considering the significance of clinical application, the goal of this study is to develop an automatic, efficient, and accurate quantitative analysis system to measure the pathological progress of pterygium. Therefore, this study proposes a system using deep learning methods to obtain the WP to measure the pathological progress of pterygium. In this study, according to the value of WP, the patients are divided into three categories: normal, pterygium to be observed (WP is <3 mm), and pterygium requiring surgery (WP is ≥3 mm). In addition, this system also measures the area of pterygium in the cornea to further realize accurate medical diagnosis. The proposed system is composed of three modules: cornea segmentation module, pterygium segmentation module, and measurement module. The main contributions of this study are as follows:

1. This study proposes a system to measure the pathological progress of pterygium. The system can automatically and efficiently process the anterior segment images input by patients and output quantitative analysis results such as the WP, the area of pterygium in the cornea, and the pathological status, to assist doctors to formulate treatment strategies and operation plans.
2. This study develops a cornea segmentation module based on U-Net model to segment the cornea in the anterior segment image. Since the segmentation results have jagged edges, according to a priori condition that the cornea is ellipse, an ellipse fitting algorithm is used to fit the original segmentation results to obtain more complete cornea.
3. This study develops a pterygium segmentation module based on the improved U-Net++ model to segment the pterygium in the cornea image output using the cornea segmentation module. In the pterygium segmentation task, the positive and negative samples are often unbalanced, and the shape and size of pterygium is different in each image. Therefore, this study improves the U-Net++ model by adding Attention gate units before each up-sampling layer, so that the improved U-Net++ model can pay more attention to the location and the shape of pterygium, which improves the performance of U-Net++ in the pterygium segmentation task.
4. This study develops a measurement module to measure the WP and the area of pterygium in the cornea. The shape of the cornea not invaded by pterygium is obtained by performing logical XOR on the outputs of two segmentation module. WP is obtained by measuring the ratio of the minimum Euclidean distance from the center of the ellipse to the edge (MD) to the Euclidean distance of the radius of the transverse axis of the ellipse (RD). The area of pterygium in the cornea is obtained by calculating the ratio of the area of pterygium to the area of the cornea in the image. According to the size of the WP (=0 or >3 mm), the patients are classified as normal, pterygium to be observed, and pterygium requiring surgery.

## Dataset Description

**Figure 1:**
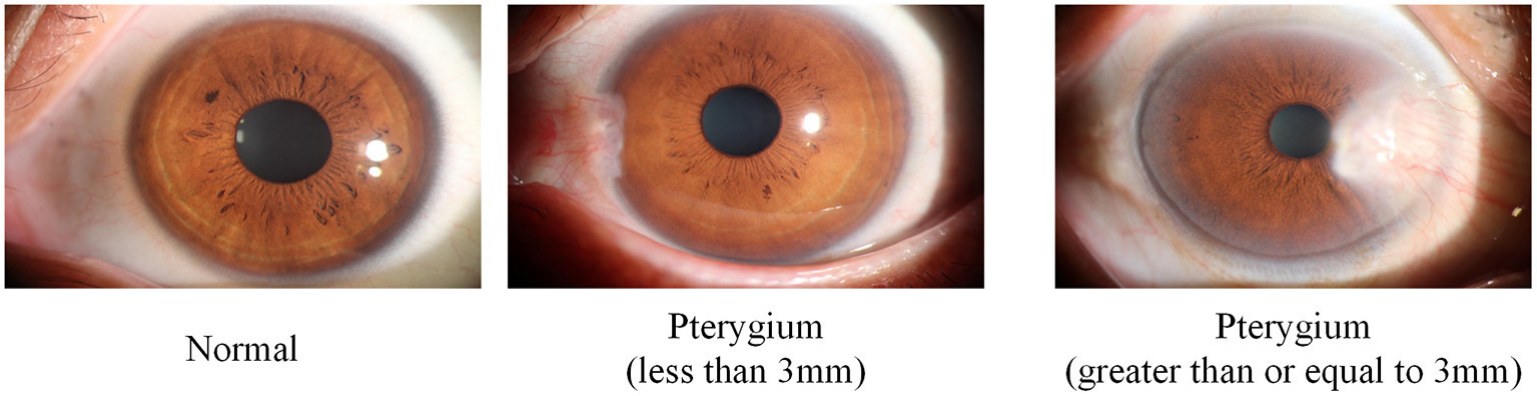
Three types of anterior segment images in the dataset.

The dataset used in this study was collected from the ocular surface diseases center at the Affiliated Eye Hospital of Nanjing Medical University. It consisted of 489 anterior segment images, including 244 normal eyes and 245 eyes with pterygium. All images were taken with a Canon SLR camera (model: Canon EOS 600D), and the slit lamp was illuminated with diffuse illumination, with a magnification of 10x and an image resolution of 5,184 × 3,456, exposure time 1/30 s. Three types of anterior segment images are shown in Figure 1. This study used LabelMe annotation software to label the dataset, including the labels of the cornea and that of the pterygium in the cornea, which were used for training and testing the segmentation models. All labels were labeled under the guidance of professional doctors, and all the segmented labels were checked and confirmed by professional doctors.

## Methods

**Figure 2:**
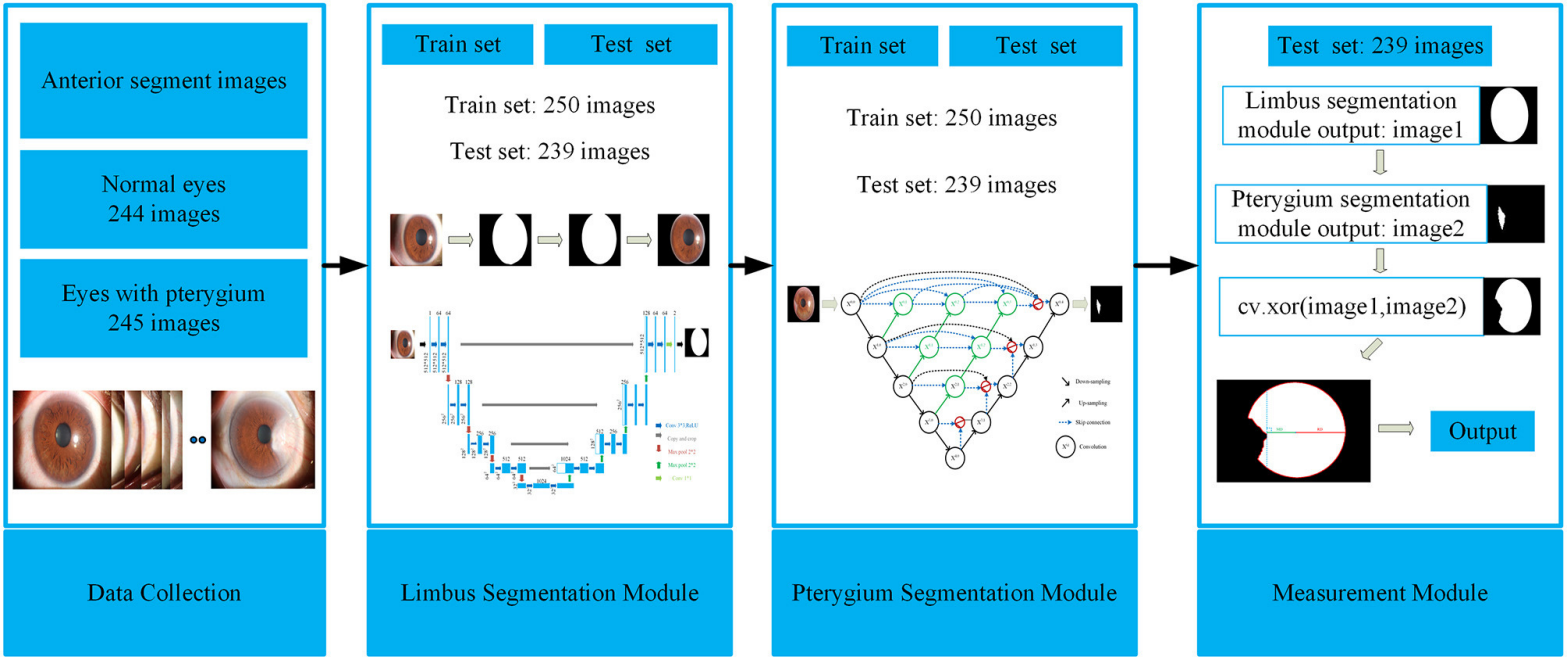
Framework of the system for measuring pathological progress of pterygium.

This study aims to develop a system that can automatically, efficiently, and accurately measure the pathological progress of pterygium by quantitatively analyzing patients' anterior segment images, as shown in Figure 2. The system is composed of three modules: a cornea segmentation module, a pterygium segmentation module, and a measurement module. The first two segmentation modules adopt the deep learning networks that have been widely used in the research of medical image segmentation in recent years. These networks segment images in an end-to-end mode, which reduces manual preprocessing and subsequent processing to improve the segmentation accuracy and the automation of the whole system. The measurement module processes the outputs of the first two modules to obtain the shape of the non-invading cornea, and then measures the ratio of MD to RD. The WP is calculated by multiplying $1-\frac{MD}{RD}$ by the transverse diameter of the cornea. Then the corresponding classification is carried out using the value of WP to measure the pathological progress of pterygium. Finally, the system outputs the pterygium's status, the WP, and the area of pterygium in the cornea to realize an accurate medical diagnosis.

### Cornea Segmentation Module

The cornea is an oval shaped transparent film located in the front of the eyeball. During the growth phase of pterygium, its head is located in the cornea, and its body is located in the iris. It is medically recognized that the pathological progress of pterygium is positively correlated with the WP. When WP is >3 mm, surgical resection is required. Considering this, the first module of the system proposed in this study is the cornea segmentation module, which aims to segment the cornea part of the input anterior segment images, so that the pterygium in the cornea can be segmented separately. The complete shape of the cornea can be obtained for the final measurement module.

#### Deep Architecture

U-Net is used in the cornea segmentation module. U-Net was first published in the 2015 Medical Image Computing and Computer Assisted Intervention Society conference (MICCAI) and was proposed by Ronneberger et al. (12). The U-Net proposal was inspired by Fully Convolutional Networks (13). It is a segmentation network proposed for medical image segmentation, and it can use a small amount of data to train a model with high edge extraction performance. Over the years, it has become widely used in the field of biomedical image segmentation (14, 15).

**Figure 3:**
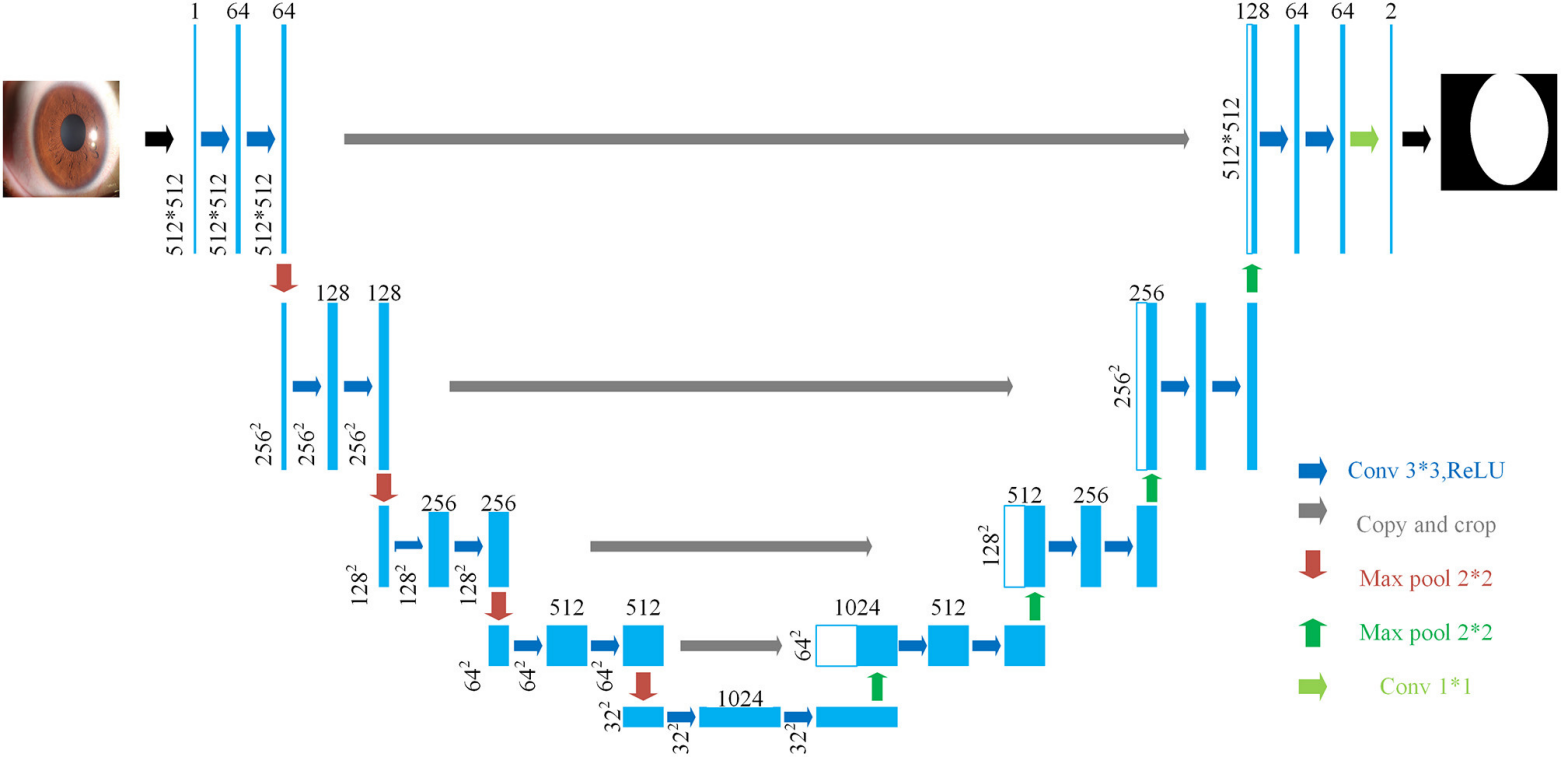
The structure of U-Net.

The structure of U-Net is shown in Figure 3. Its encoder uses four consecutive down-sampling to obtain the feature map of each input image. Its decoder correspondingly uses four consecutive up-sampling to restore the feature map with high-level semantics obtained by the decoder to a feature map with the resolution of the original image. Then the model outputs the result through a Sigmoid function to complete the semantic segmentation of the original image. While up-sampling, U-Net uses skip connection for each up-sampling layer, instead of directly propagating forward and backward on high-level semantic features, ensuring that the final feature map obtained by decoding integrates multiscale feature information and makes the final segmentation result more accurate.

#### Training Details

There are 489 anterior segment images in the dataset, including 244 normal eyes and 245 eyes with pterygium. The dataset is divided into a training set and a test set. The training set has a total of 250 images, including 123 normal eyes and 127 eyes with pterygium. The test set has a total of 239 images, including 121 normal eyes and 118 eyes with pterygium. To better meet the image training standard of convolutional neural networks, this study uses an interpolation method based on regional pixel relations to reduce the resolution of each image to 512 × 512.

In the preprocessing stage, this study uses image contrast stretching and adaptive image equalization methods to enhance the contrast between the cornea and the background in the anterior segment image. To avoid the over-fitting phenomenon during training, enhance the generalization performance of the network, and improve the segmentation accuracy of the network, strategies such as flipping, translation, and rotating are used to augment the training set.

During the training process, we set the Binary Cross Entropy with Logits Loss (BCE With Logits Loss) as the loss function for network training. The BCE With Logits Loss function combines the Sigmoid layer and Binary Cross Entropy Loss (BCE) (16), which means that it automatically performs Sigmoid processing on the output first, then performs binary cross entropy calculation on the output and the ground truth. Assuming there are N batches, *x*_*n*_is the predicted value of the *n*_th_ batch, *y*_*n*_ is the ground truth of the *n*_th_ batch, and *l*_*n*_ is the loss value of the *n*_th_ batch. The BCE With Logits Loss function is as follows:


(1) $$\begin{array}{r} \begin{array}{r} \text{Loss}\text{ = \{}l_{1}\text{, . . . , }l_{N}\text{\} , }l_{n}\text{ = }-\text{(}y_{n}\text{ log (}\sigma \text{ (}x_{n}\text{))}\\ \text{+(}1\;-\;y_{n}\text{) log (}1\text{ - }\sigma \text{ (}x_{n}\text{)))} \end{array} \end{array}$$

σ(*x*_*n*_) is the Sigmoid function, and *x* can be mapped to the interval (0, 1) ,as shown below:


(2) $$\begin{array}{l} \sigma \left(x_{n}\right)\;=\;\frac{1}{1\;+\;e^{-x}} \end{array}$$

Setting RMSprop (17) as the optimizer for network training, the initial learning rate was 1e−5, the first and second momentum decay rates were set to 0.9 and 0.99, batch size was set to 4, and the training was adjusted by L2 weight decay with a weight decay rate of 1e−8. The network was trained for 100 epochs, and 10% of the training set is randomly selected as the validation set. The Dice coefficient of the validation set is regarded as the indicator for selecting the optimal segmentation model. The Dice coefficient is usually used to calculate the similarity between two samples. The calculation method for the Dice coefficient (18) is shown below:


(3) $$\begin{array}{l} \text{Dice}\;=\;\frac{2|X\cap Y|}{\left|X|\;+|Y\right|} \end{array}$$

X is the predicted result of the image to be segmented, Y is the ground truth of the image to be segmented, and (0, 1) is the value range of Dice coefficient.

#### Subsequent Processing

When using U-Net for target segmentation, jagged edges in the edge area exist. Therefore, according to the prior knowledge that the physical shape of the cornea is an ellipse, the least squares method is used to fit the ellipse with the smallest algebraic distance between the segmentation boundary points (19) and to realize the smooth fitting of the segmentation results, then the final cornea segmentation results are obtained.

To obtain the training set and test set of the next module, in the training set of this module, the ground truth was used to extract the cornea area in the anterior segment image, and 250 images containing only the cornea are obtained. In the test set, the segmentation results of the test set output by the cornea segmentation module are used to extract the cornea in the anterior segment image of the test set, and 239 images containing only the cornea are obtained.

### Pterygium Segmentation Module

The function of the pterygium segmentation module is to segment the pterygium in the cornea image output using the cornea segmentation module. Then input the complete pterygium shape into measurement module.

#### Deep Architecture

U-Net++ is an improved network of U-Net proposed by Zhou et al. (20) in 2020. On the basis of the original four up-sampling layers of U-Net, U-Net++ adds up-sampling to each down-sampling layer. It uses skip connection to splice the features of each layer, which solves the back propagation problem of U-Net++ at the same time. U-Net++ combines shallow features and deep features better than U-Net by adding multi-layer up-sampling and skip connections to achieve better segmentation performance. In the past years, it has also been widely used in the research of biomedical image segmentation (21, 22).

**Figure 4:**
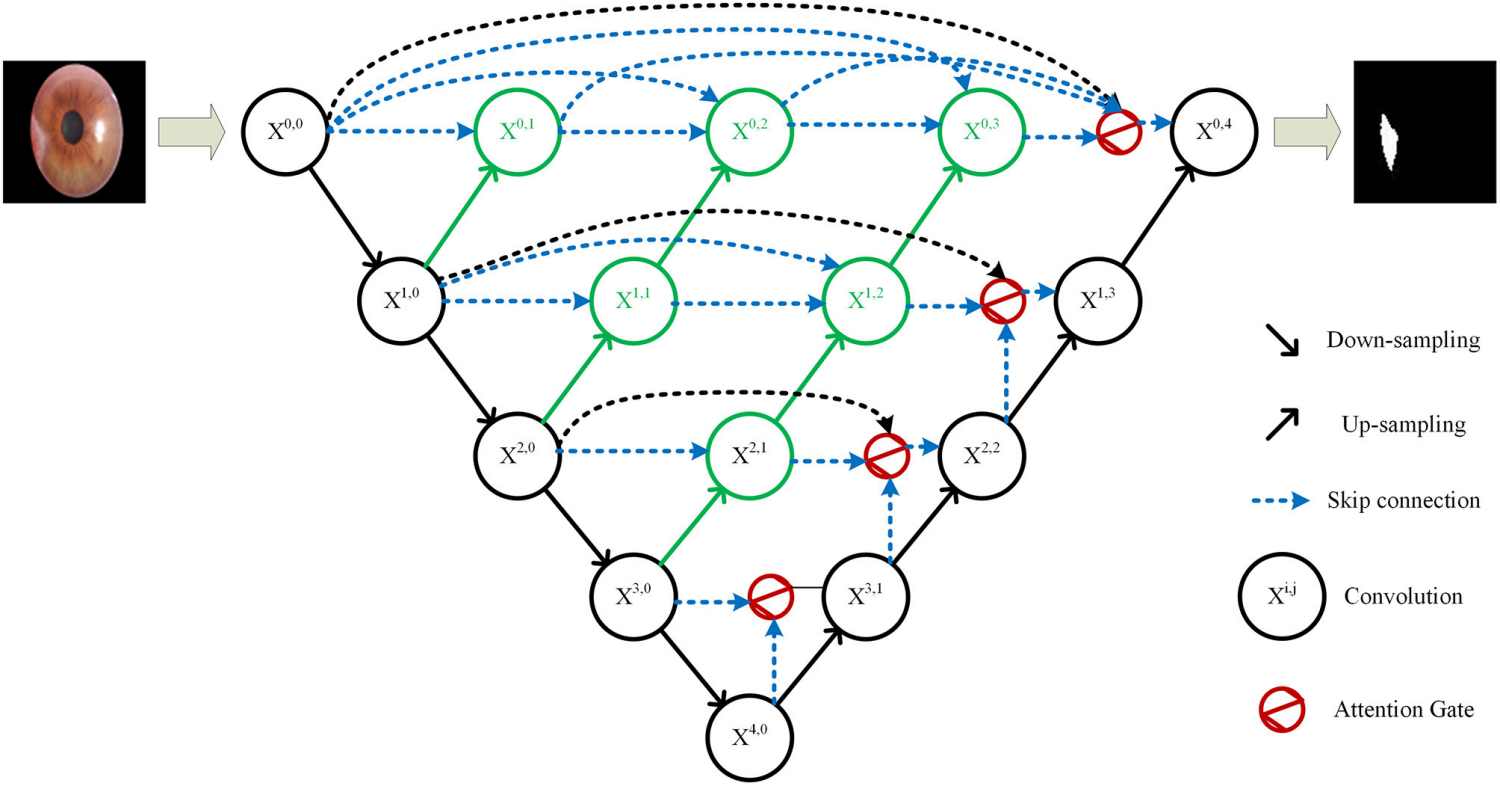
Structure of the improved U-Net++.

Since the pterygium only occupies a small area in the cornea image, the positive and negative samples in the pterygium segmentation task were unbalanced, and because of the uncertainty of the growth position of pterygium and the diversity of the shape and size of pterygium, the deep network needs to pay more attention to pterygium area, instead of background. Therefore, this study proposes an improved U-Net++ by adding Attention gates (23) before each up-sampling layer. The structure of the improved U-Net++ is shown in Figure 4.

**Figure 5:**
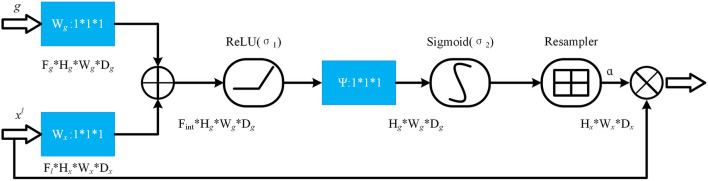
The structure of attention gate.

The specific structure of the Attention gate is shown in Figure 5. *g* is the gating signal and ***x***^***l***^ is the feature map of the upper layer. *g* comes from the next layer; hence, the size of *g* is half of the previous layer, which means that the previous layer needs to be down-sampled. ***x***^***l***^ and *g* are input to the attention module to get the weight coefficient ***a***, and then ***a*** is multiplied by ***x***^***l***^ for output. The number or channels of ***x***^***l***^ and *g* changes in the attention module from ***F***_*g*_ to ***F***_***int***_, and then to 1. In this process, *g* and ***x***^***l***^ are multiplied by the weight matrix, respectively. The weight matrix can be learned through back propagation to obtain the importance of each element of *g* and ***x***^***l***^, so the Attention gate can allow the network to learn the importance between each element and the target.

In the Attention gate of the improved U-Net++, the features of the next level and the same level are used to supervise the features of upper level, and the activation part of the network is concentrated on the area to be segmented, so that the activation value of background is reduced to optimize the segmentation.

#### Training Details

The training set in this module consists of 250 cornea images, including 123 normal eyes and 127 eyes with pterygium. The test set is derived from the cornea images output by the cornea segmentation module and a total of 239 images, including 121 normal eyes and 118 eyes with pterygium. To speed up the training speed of network, the resolution of all images is reduced to 96 × 96 through the interpolation method based on regional pixel relations.

In the pre-processing stage, the adaptive contrast stretching method is used to pre-process the images of the training set to enhance the contrast between the pterygium and the background. To avoid the over fitting of the network, improve the generalization performance of the network, and enhance the segmentation performance of the network, the images of the training set are augmented by strategies such as rotation, translation, and flipping.

During the training process, we set BCE Dice Loss (24) as the loss function of network training. BCE Dice Loss combines the BCE Loss and the Dice Loss. The Dice Loss (25) uses the Dice coefficient as a measure of loss. The Dice coefficient is usually used to calculate the similarity between two samples. The calculation method for the Dice coefficient is shown in (3). The DiceLoss is calculated as shown in (4), which is usually used in the case of unbalanced samples. The function of the BCE Dice Loss is shown in (5). Then we set Adam as the optimizer of network training, the initial learning rate was 1e−3, the first and second momentum decay rates were set to 0.9 and 0.99, batch size was set to 16, and the process of training was adjusted by L2 weight decay with a weight decay rate of 1e−4. The network was training for 300 epochs. The Mean Intersection Over Union (mIOU) of the validation set is regarded as the indicator for selecting the final segmentation model. The mIOU calculates the ratio of the intersection between the ground truth and the predicted image, as shown in (6). Among them, ***p***_***ij***_ represents the number of samples with true value of ***i***, but predicted to be ***j***. ***k*****+*1*** is the number of categories. ***p***_***ii***_ is the number of samples which are correctly predicted. ***p***_***ji***_ represents the number of samples with true value of ***j***, but predicted to be ***i***.


(4) $$\begin{array}{lll} \text{DiceLoss}\; & = & \;1\;-\;\text{Dice}, \end{array}$$

(5) $$\begin{array}{rcl} \text{BCEDiceLoss}\; & = & \;1e-3\left\{l_{1},\ldots ,l_{N}\right\}-\text{Dice},\\ l_{n}\; & = & \;-\left(y_{n}\log\left(x_{n}\right)\;+\left(1\;-\;y_{n}\right)\log\left(1\;-\;x_{n}\right)\right), \end{array}$$

(6) $$\begin{array}{lll} \text{mIOU}\; & = & \;\frac{1}{k+1}\overset{k}{\underset{i=0}{\sum }}\frac{p_{ii}}{\overset{k}{\underset{j=0}{\sum }}p_{ij}+\overset{k}{\underset{j=0}{\sum }}p_{ji}-p_{ii}} \end{array}$$

#### Subsequent Processing

The results of segmentation network usually have jagged edges in the edge area. Therefore, in this module, after the network is trained and output the segmentation results, the edge of segmented pterygium needs to be smoothed.

### Measurement Module

The purpose of the measurement module is to quantitatively measure the shape of the intact cornea, calculate the WP and the area of pterygium in the cornea, then the pathological progress of pterygium can be judged by analyzing the WP.

The first step is to obtain 239 complete cornea images output by the cornea segmentation module and 239 pterygium images output by the pterygium segmentation module, then enlarge all images to 512 × 512 to ensure that the shape of the cornea in all images is same. After that, the pixel-by-pixel logical XOR is performed on the output images of two segmentation modules to obtain the cornea images with the pterygium removed.

**Figure 6:**
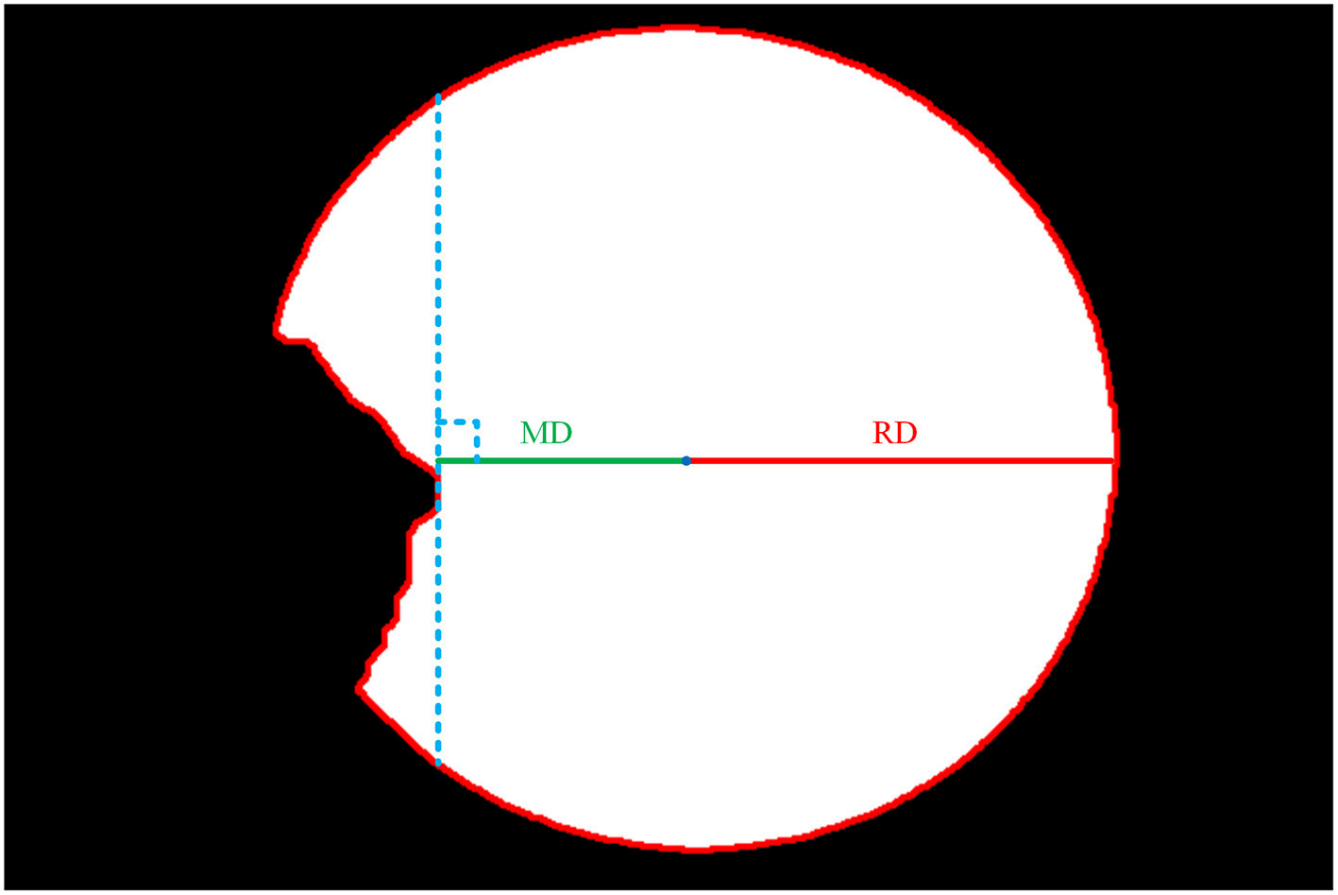
Cornea images not invaded by pterygium.

The second step is to extract the edge of the cornea images output by the cornea segmentation module to obtain the boundary contour of cornea. We obtain the ellipse center coordinates by measuring the intersection of the longest axis in the X and Y direction of the ellipse and obtain the Euclidean distance of the radius at the transverse axis (RD), as shown in Figure 6.

The third step is to extract the edge of the intact cornea images obtained in the first step, then calculate the minimum Euclidean distance from the center of the ellipse to the edge (MD) using the center coordinates obtained in the second step, as shown in Figure 6.

The final step is to calculate the ratio of MD and RD, then calculate the WP according to the transverse diameter of the cornea, as shown in (7). According to the previous study, there is a variation ranging from 10.7 to 12.58 mm of the transverse diameter of the cornea (26). The actual diameter of the cornea can be measured instrumentally. Because of the lack of actual diameter of cornea, and in order to ease the calculation, this study assumes that the transverse diameter of the cornea is 11.5 mm.


(7) $$\begin{array}{r} WP\;=\;\frac{\text{diameter}}{2}\;\times \;\left(1-\frac{MD}{RD}\right) \end{array}$$

To ensure the accuracy of segmentation and the effectiveness of the system in practical application, this study considers that there may be images of normal eyes in practice, so the dataset also contains the anterior segment images of normal eyes. Therefore, the system proposed in this study divides the input anterior segment images into three categories according to WP size (0 or >3 mm): normal (WP is 0), pterygium to be observed (WP is >0 and <3 mm), pterygium requiring surgery (WP is ≥3 mm).

The measurement module also calculates the ratio of the pixel area of pterygium (AP) to the pixel area of the cornea (AC) and multiplies it by the actual area of the cornea to obtain the actual area of the pterygium in the cornea, as shown in (8). As mentioned above, the diameter of the cornea is ranging from 10.7 mm to 12.58 mm. Because of the lack of actual diameter of cornea, this study assumes that the cornea is a horizontal circle with a diameter of 11.5 mm to calculate the actual area of the cornea.


(8) $$\begin{array}{l} \text{Area}\;\mathrm{of}\;\text{pterygium}\;=\;\pi \times {\left(\frac{\text{diameter}}{2}\right)}^{2}\times (\frac{AP}{AC}) \end{array}$$

To sum up, this study proposes a system for measuring the pathological progress of pterygium using deep learning. Using an end-to-end learning method, only the anterior segment image of the patient is required as input to determine what category the patient is in. When the patient is judged to be a patient with pterygium, the actual value of WP and the area of pterygium can also be output, which avoids the situation that WP can only be estimated by doctors' experience because of lack of professional measurement tools; hence, accuracy of diagnosis is significantly increased.

## Results

The WP measured in the measurement module can be used to judge the pathological progress of pterygium. Therefore, this study establishes a complete system for measuring pterygium's process by developing a cornea segmentation module, a pterygium segmentation module and a measurement module.

### Segmentation Results of the Cornea Segmentation Module

**Figure 7:**
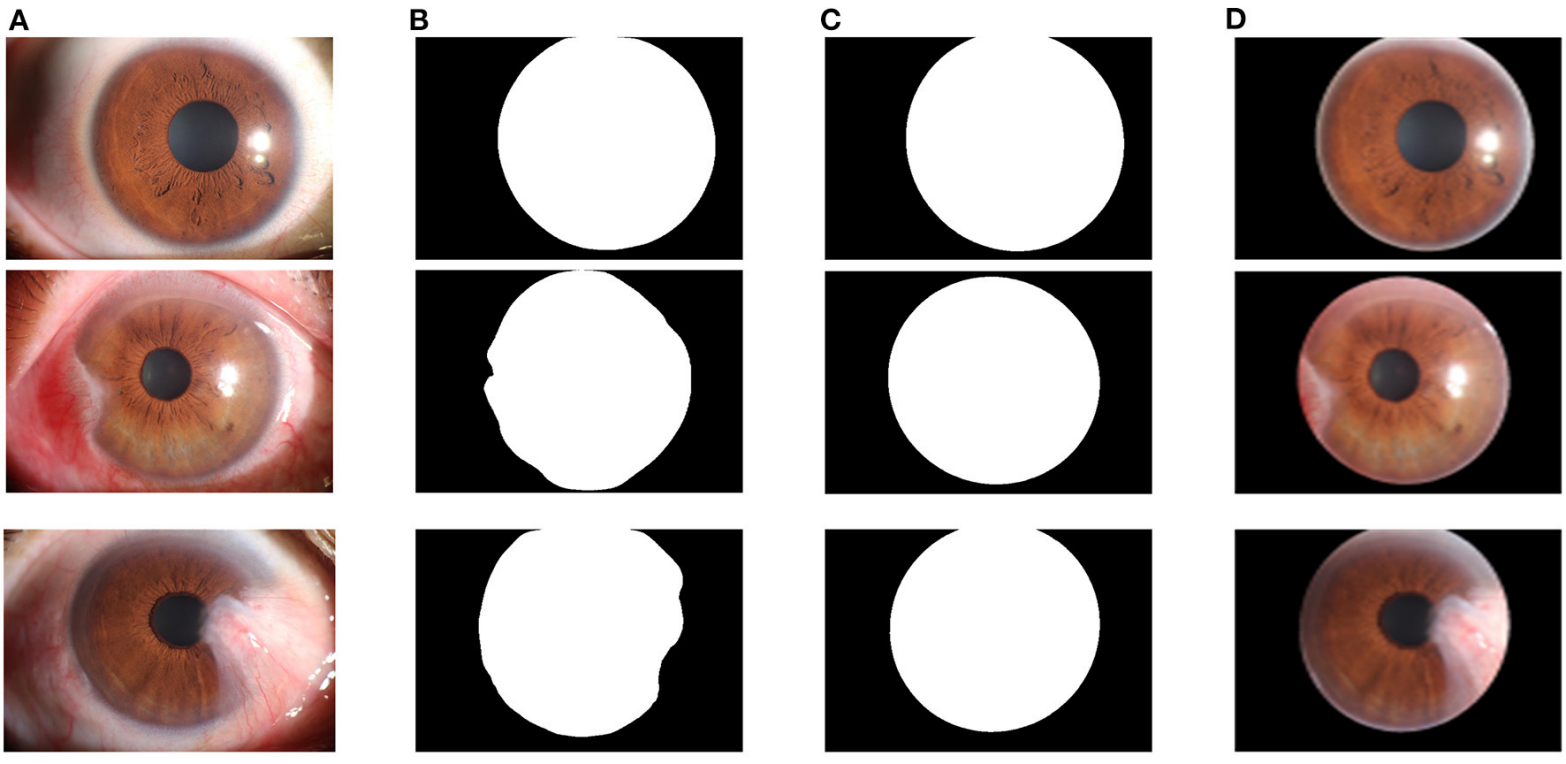
Segmentation results of the cornea segmentation module: **(A)** Original anterior segment images. **(B)** Segmentation results of the trained U-Net model. **(C)** Output results with smooth edge after ellipse fitting. **(D)** Final cornea images output by the cornea segmentation module.

The core of the cornea segmentation module is the U-Net. Two hundred and fifty images are used in the training process, and the performance of the network is tested on 239 images. According to the abovementioned workflow, the U-Net is trained using the training set, and the model with the best Dice coefficient in the validation set is obtained as the segmentation model of the cornea segmentation module. Then we test the performance of the model on the test set to evaluate its performance in practical applications. Figure 7A shows the visual samples of the original anterior segment images. Figure 7B shows the visual samples of the segmentation results of the cornea segmentation module on the test set, including the three categories: normal, pterygium to be observed, and pterygium requiring surgery.

To quantitatively evaluate the performance of the U-Net model in the cornea segmentation module, the Dice coefficient and the mIOU are calculated using the test set. The function for the Dice coefficient is shown in Equation (3). The function for the mIOU is shown in Equation (6). The Dice coefficient of the final output model on the test set was 0.9620, and the mIOU was 0.9338.

Since the cornea segmentation results directly output by the network have jagged edges, the least squares ellipse fitting is used to smoothly fit the segmentation results to obtain a complete cornea shape, as shown in Figure 7C. To obtain the cornea images required for the next module for pterygium segmentation, the cornea segmentation results are used to extract the cornea in the original anterior segment images, as shown in Figure 7D.

### Segmentation Results of the Pterygium Segmentation Module

**Figure 8:**
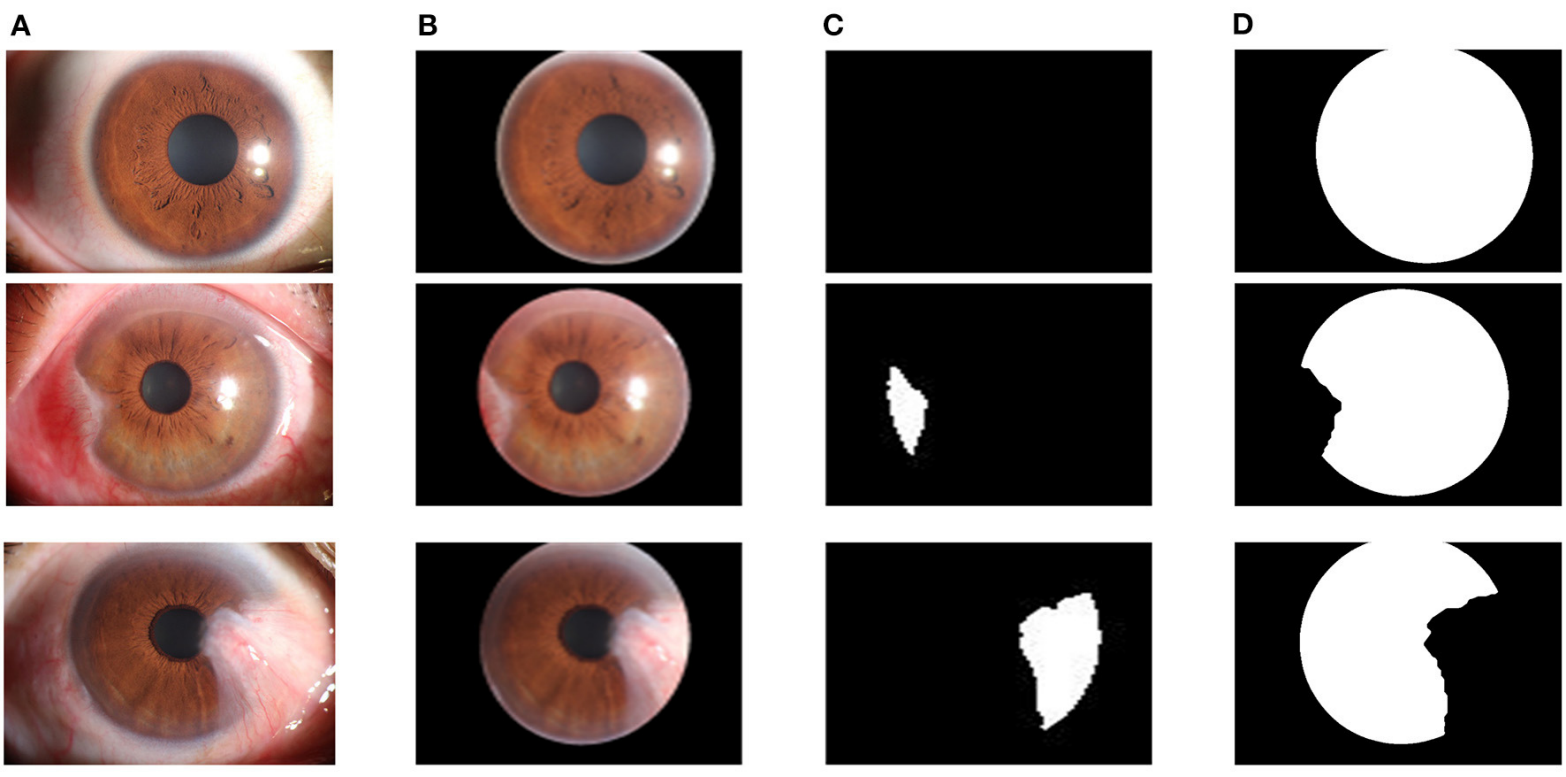
Segmentation results of the pterygium segmentation module: **(A)** The original anterior segment images. **(B)** The cornea images output by the cornea segmentation module. **(C)** The segmentation results output by the improved U-Net++. **(D)** The cornea not invaded by pterygium.

In the pterygium segmentation module, considering the diversity of the shape and size of pterygium in the pterygium segmentation task, this study improves U-Net++ by adding the Attention gate before each up-sampling layer to extract the information related to pterygium. According to the abovementioned workflow, the improved U-Net++ was trained, and the model with the best mIOU on the validation set was selected as the segmentation model in the pterygium segmentation module. We tested the performance of the model on the test set to evaluate its performance in practical applications. Figure 8A shows the visual samples of the original anterior segmented images. Figure 8B shows the visual samples of the segmentation results of the cornea images output by the cornea segmentation module. Figure 8C shows the visual samples of the segmentation results of the pterygium segmentation module on the test set, including the three categories: normal, pterygium to be observed, and pterygium requiring surgery. Figure 8D shows the obtained cornea shape without the pterygium, which will be output to the next module to measure the WP.

**Figure 9:**
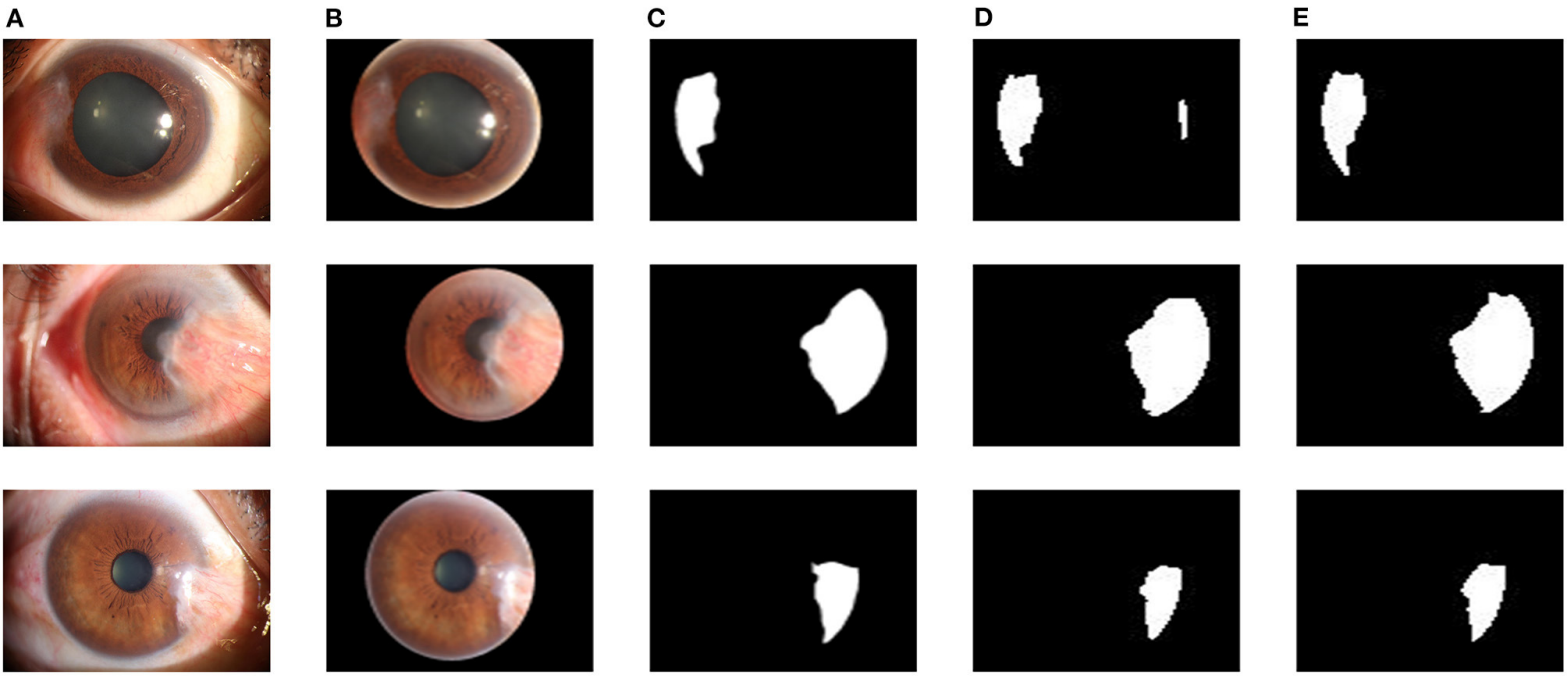
Segmentation results of two models: **(A)** The original anterior segment images. **(B)** The cornea images output by the cornea segmentation module. **(C)** Ground truth. **(D)** The segmentation results of the U-Net++ model. **(E)** The segmentation results of the improved U-Net++ model.

The improved U-Net++ is used in the pterygium segmentation module, and the Attention gate is added before each up-sampling layer. To quantitatively evaluate the segmentation performance of the improved U-Net++, the Dice coefficient and mIOU of the test set are calculated. To verify the effectiveness of the Attention gate's joining, a comparative experiment is conducted. This section uses the same training set to train the original U-Net++, output the optimal model of mIOU, and verify the performance on the test set. Figure 9 shows the segmentation results of the U-Net++ model and the improved U-Net++ model. It can be seen that the improved U-Net++ model proposed in this study has a better segmentation effect on the edge details of the pterygium and can segment a more complete pterygium shape than the original U-Net++. Table 1 shows the quantitative performance indicators of the two models on the test set. It can be seen that when Accuracy (ACC) are similar, the method proposed in this study is superior to the original U-Net++ in terms of mIOU and Dice coefficient indicators.

**Table 1:** Quantitative performance indicators of two models.

**Model**	**ACC**	**Dice**	**mIOU**
U-Net++	0.9901	0.8678	0.8644
The proposed model	**0.9905**	**0.9020**	**0.8944**

*ACC is the accuracy, Dice is the Dice coefficient, mIOU is the Mean Intersection Over Union*.

### Results of Measurement Module

Table 2 shows the measurement results of the WP of 15 samples of the test set. Zero, 1, and 2 under the CLASS heading represent normal eye, patient with pterygium to be observed, and patient with pterygium requiring surgery, respectively.

**Table 2:** Measurement result samples.

**Images**	**RD**	**MD**	**WP (mm)**	**CLASS**	**Area (mm^**2**^)**
027.PNG	208	208	0	0	0
029.PNG	204	204	0	0	0
033.PNG	208	208	0	0	0
035.PNG	208	208	0	0	0
036.PNG	208	208	0	0	0
076.PNG	212	51	4.17	2	8.95
079.PNG	212	64	3.83	2	9.88
080.PNG	212	86	3.26	2	9.14
083.PNG	196	29	4.68	2	10.37
086.PNG	208	60	3.91	2	15.27
116.PNG	196	145	1.43	1	1.86
119.PNG	208	165	1.13	1	1.72
120.PNG	212	128	1.87	1	4.14
124.PNG	216	162	1.37	1	1.55
152.PNG	204	3	5.41	2	18.50

## Discussion

### Discussion of Segmentation Results

The performance of the two segmentation modules is qualitatively evaluated by obtaining the segmentation images, and is quantitatively evaluated by the Dice coefficient, the mIOU and the ACC. In the cornea segmentation module, the Dice coefficient and mIOU of the trained U-Net model on the test set are 0.9620 and 0.9338, respectively. Through visual inspection, it is found that the segmentation results directly output by the U-Net model have jagged edges. In the subsequent processing, the least square ellipse fitting is used to obtain a more complete cornea. In the pterygium segmentation module, to verify the effectiveness of the improved U-Net++ model, a comparative experiment is conducted. The obtained Dice coefficient, mIOU, and ACC of the U-Net++ model on the test dataset were 0.9020, 0.8944, and 0.9905, respectively, and the Dice coefficient, mIOU, and ACC of the improved U-Net++ model on the test dataset were 0.8678, 0.8644, and 0.9901, respectively. It can be seen that under the condition that the ACCs are similar, the mIOU and the Dice coefficient of the improved U-Net++ model are 3 and 3.42% higher than that of the original U-Net++ model, respectively, which proves the effectiveness of the improvement.

### Discussion of Measurement Results

In the measurement module, the actual WP is calculated using Equation (7) and the area of pterygium in the cornea is calculated using Equation (8). Table 2 shows some samples of measurement results of the measurement module on the test set, including the WP, patient's pterygium pathological status, and the area of pterygium in the cornea.

To verify the accuracy of the final predicted results of the system proposed in this study, we use chi-square test and kappa consistency test to qualitatively compare the difference and consistency between the predicted results of the system proposed in this study and that obtained from a doctor's visual inspection. Chi-square test (27) is a statistical method to test the difference (whether it is related) between two categorical variables. The basic idea of chi-square test is as follows: first propose the null hypothesis H_0_, that is, the two categorical variables are not correlated; second calculate the chi-square value χ^2^ based on this premise; third determine the null hypothesis based on the chi-square distribution and degrees of freedom (df) under the circumstances; final the more extreme probability P are obtained. If the *P* < 0.005, it means that the two variables are strongly correlated, and the null hypothesis is rejected, there is a significant correlation between the two variables. To quantitatively measure the correlation of two variables, that is, the degree of correlation between the system's predicted results and the doctor's visual inspection results, this study introduces the column connection number, C, and its calculation formula is as follows:


(9) $$\begin{array}{l} C\;=\;\sqrt{\frac{\chi^{2}}{n+\chi^{2}}} \end{array}$$

where *n* is the sample size.

Kappa consistency test (28) is usually used to investigate whether different diagnostic methods have consistency in diagnostic results. The original hypothesis of kappa consistency test is that there is no significant consistency between the two diagnostic results. Based on this premise, kappa consistency coefficient and progressive significance P are calculated. If the *P* < 0.005, the original hypothesis is rejected, and the two diagnostic results are significantly consistent. The kappa consistency coefficient is a measure of the consistency between two categories, based on linear weighting, the formula is as follows:


(10) $$\begin{array}{l} P_{o}\;=\frac{\overset{k}{\underset{i=1}{\sum }}p_{ii}}{\overset{k}{\underset{i=1}{\sum }}\overset{k}{\underset{j=1}{\sum }}p_{ij}}, \end{array}$$

(11) $$\begin{array}{l} P_{e}\;=\frac{\overset{k}{\underset{i}{\sum^{\text{​}}}}(\overset{k}{\underset{j}{\sum^{\text{​}}}}p_{ij}\times \overset{k}{\underset{j}{\sum^{\text{​}}}}p_{ji})}{{\left(\overset{k}{\underset{i=1}{\sum^{\text{​}}}}\overset{k}{\underset{j=1}{\sum^{\text{​}}}}p_{ij}\right)}^{2}} \end{array}$$

The kappa consistency coefficient is a function of two quantities. Here, ***p***_***ij***_ is the element in row *i* and column *j* in the crosstab, and *k* is the number of categories.

The kappa consistency coefficient is given by,


(12) $$\begin{array}{l} \text{kappa}\;=\;\frac{P_{o}+P_{e}}{1-P_{e}} \end{array}$$

Table 3 is a cross table of the predicted results and doctor's visual inspection results, where 0, 1, and 2 represent normal eye, patient with pterygium to be observed, and patient with pterygium requiring surgery, respectively. It can be seen that the predicted results are completely consistent with the doctor's visual inspection results when the patient is normal. In the case of patients with pterygium, the predicted results and the doctor's visual inspection results are different for some patients.

**Table 3:** Crosstab of the predicted results and the doctor's visual inspection results.

**Prediction results**					
**VisualInspectionResults**		**0**	**1**	**2**	**Total**
	**0**	121	0	0	121
	**1**	0	68	8	76
	**2**	0	4	38	42
**Total**		121	72	46	239

We input the content of the crosstab into SPSS for chi-square test and kappa consistency test. The results of the chi-square test are shown in Table 4. χ^2^ = 386.233, df = 4, *P* < 0.0001, and the H_0_ is rejected, that is, the predicted results are correlated with the doctor's visual inspection results. The calculated column connection number is 0.786, and the maximum value of the column connection number in the 3 × 3 column connection table is known to be 0.8165, which means that there is a strong relationship between the predicted results and the doctor's visual inspection results. The results of the kappa consistency test are shown in Table 5. *P* < 0.0001, which means that the null hypothesis is rejected, that is, the predicted results are significantly consistent with the doctor's visual inspection results. The Kappa consistency coefficient is 0.918, which is >0.8. It can be considered that there is a high consistency between the predicted results and the doctor's visual inspection results.

**Table 4:** Results of chi-square test.

	**df**	***P***	**H_**0**_**	**C**
386.233	4	<0.0001	Reject	0.786

**Table 5:** Results of Kappa consistency test.

**Kappa**	***P***	**H_**0**_**
0.918	<0.0001	Reject

It can be seen from Table 3 that when the image of a normal patient is input, the predicted result of the system is completely consistent with the doctor's visual inspection results. But when inputting images of patients with pterygium, the predicted results and the doctor's visual inspection results are different for some patients. To quantitatively analyze the difference, we use the doctor's visual inspection results as the ground truth, and we use Receiver Operating Characteristic (ROC) curve, Area Under ROC Curve (AUC) value, Accuracy, Specificity, Sensitivity, Precision, and F1-score (29) to analyze the binary classification of patients with pterygium. The above indicators are based on four measurement values, namely True Positive (TP), True Negative (TN), False Positive (FP), and False Negative (FN). In this task, Positive stands for “Pterygium requiring surgery,” and Negative stands for “Pterygium to be observed.”

Accuracy reflects the ratio of the number of correctly detected samples to the number of all samples.


(13) $$\begin{array}{l} \text{Accuracy}\;=\;\frac{TP+TN}{TP+TN+FP+FN} \end{array}$$

Specificity reflects the proportion of correctly detected negative samples in all relevant samples. In this study, it reflects the consistency between the predicted results and the doctor's visual inspection results for the images of patients with pterygium to be observed.


(14) $$\begin{array}{l} \text{Specificity}\;=\;\frac{TN}{TN+FP} \end{array}$$

Sensitivity reflects the proportion of positive samples that are correctly detected in all relevant samples. In this study, it reflects the consistency between the predicted results and the doctor's visual inspection results for the images of patients with pterygium requiring surgery.


(15) $$\begin{array}{l} \text{Sensitivity}\;=\;\frac{TP}{FN+TP} \end{array}$$

Precision Rate (PR) reflects the probability that the patient detected as a Positive sample is actually a Positive sample.


(16) $$\begin{array}{l} PR=\frac{TP}{FP+TP} \end{array}$$

F1-score considered both Precision Rate and Recall Rate at the same time, which is expressed as the harmonic average of the two. When the sample is unbalanced, it has a better evaluation effect.


(17) $$\begin{array}{l} F1=2\times \frac{PR\times \text{Sensitivity}}{PR+\text{Sensitivity}} \end{array}$$

To compare the prediction performance of the system from different aspects, this study draws the ROC curve based on the Specificity and Sensitivity values corresponding to the different WP classification threshold. The ordinate is Sensitivity, and the abscissa is 1-Specificity. According to the drawn ROC curve, the AUC value can be calculated, which is used to measure the prediction performance of the system.

**Figure 10:**
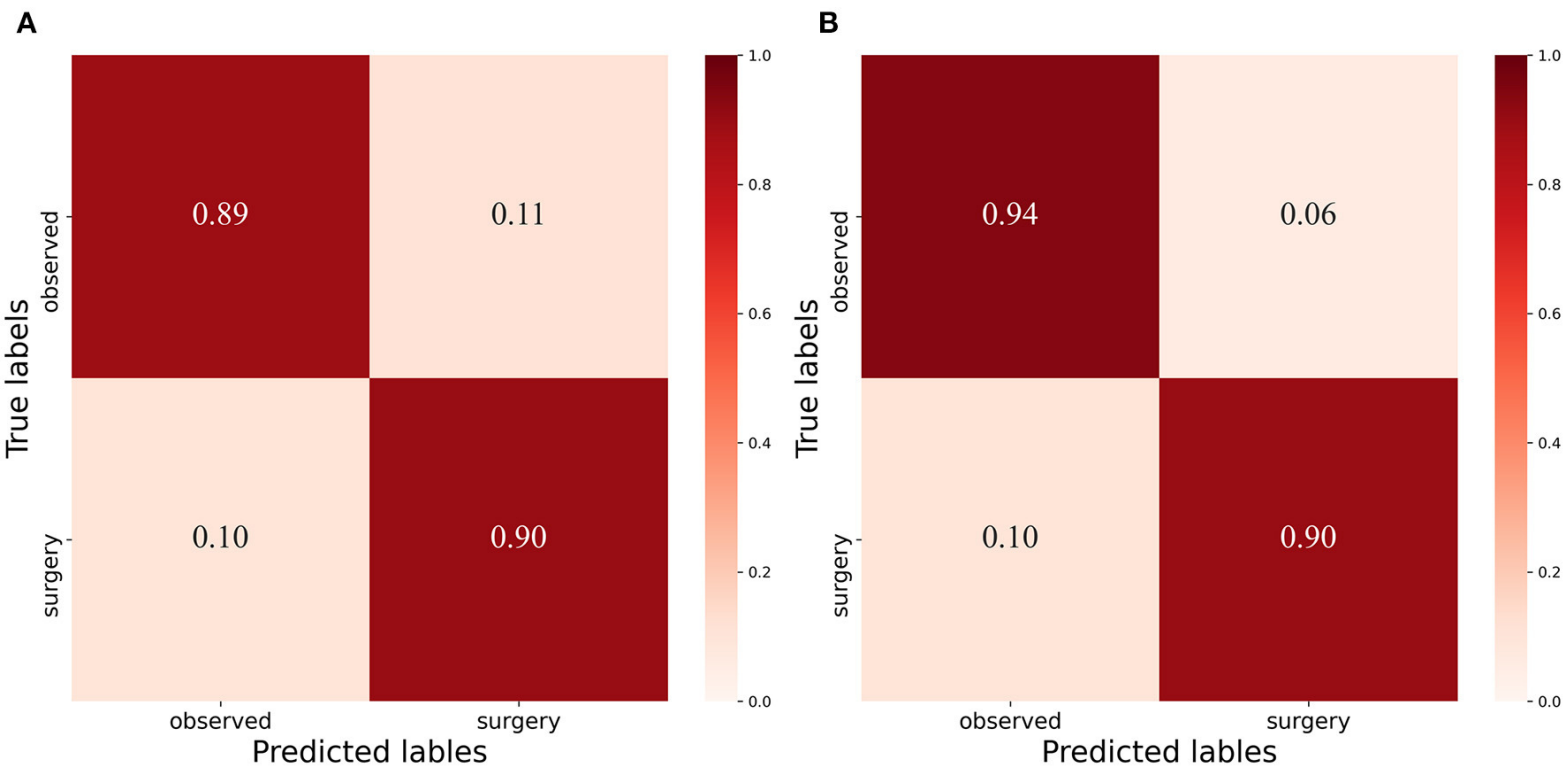
Confusion Matrix: **(A)** Confusion matrix of binary classification task with threshold 3mm. **(B)** Confusion matrix of binary classificat ion task with threshold 3.17mm.

**Figure 11:**
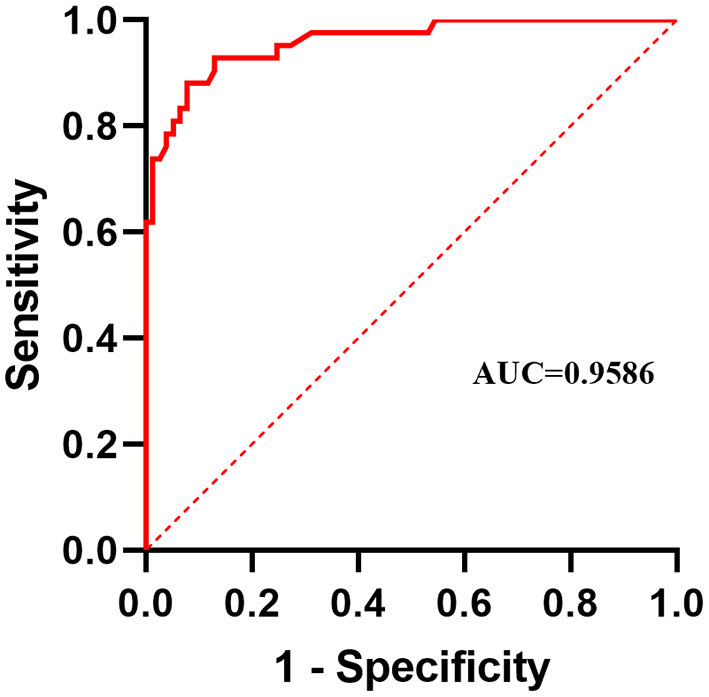
Receiver operating characteristic curve.

The confusion matrix between the predicted result and the doctor's visual inspection for images of patients with pterygium is calculated as shown in Figure 10A. The metrics of the binary classification task are as shown in Table 6. Taking the WP as the test variable, and the doctor's visual inspection result as the state variable, the ROC curve obtained is shown in Figure 11, and the AUC obtained is 0.9856.

**Table 6:** Summary of the prediction results.

**Threshold (mm)**	**Accuracy**	**Specificity**	**Sensitivity**	**PR**	**F1**
3	0.8983	0.8831	**0.9048**	0.8261	0.8636
3.17	**0.9237**	**0.9351**	0.9024	**0.8809**	**0.8916**

It can be seen that the Specificity and Sensitivity values are both ~0.9. The AUC value of the ROC curve with the WP as the test variable is 0.9586, which means when the critical threshold of WP is selected as 3 mm, it is not the optimal critical threshold. To find the optimal critical threshold, we calculate the maximum Youden index of the ROC curve, and the corresponding classification threshold is 3.17 mm, that is, the optimal critical threshold of WP is 3.17 mm, which is different from the threshold selected in this study. Then we set the classification threshold to 3.17 mm to obtain new predicted results, and the indicators of the predicted results are shown in Table 6. The confusion matrix with threshold 3.17 mm is calculated as shown in Figure 10B. It can be seen that when the threshold is set to 3.17 mm, the Accuracy, Specificity, PR, and F1-score are higher than the indicators obtained when the threshold is set to 3 mm by 2.54, 5.2, 5.48, and 2.8%, respectively. This is because the default transverse diameter of the cornea when calculating the WP is 11.5 mm in this study, but in real life, the transverse diameter of the cornea of different patients is between 10.7 and 12.58 mm (26). To compensate for this, the system proposed in this study provides options that can be modified, that is, clinicians can input the actual transverse diameter of the cornea to obtain a more accurate WP and predicted results. When calculating the area of the pterygium in the cornea, the average value of the actual vertical diameter and the actual transverse diameter of the cornea can be calculated as the diameter of the circle for calculating the area of the cornea, to obtain a more accurate result.

After observing the samples whose predicted results are inconsistent with the doctor's visual inspection results, we find that when the WP is about 3 mm, the WP cannot be accurately measured manually, which leads to misjudgment of visual inspection results, as shown in Table 7. The doctor's visual inspection result in the first row of is the “Pterygium requiring surgery” category, the WP measured by the system is 2.56 mm, and the system's predicted result is the “Pterygium to be observed” category; The doctor's visual inspection result of the second row is the “Pterygium requiring surgery” category, the WP measured by the system is 2.67 mm, and the system's predicted result is “Pterygium to be observed” category; The doctor's visual inspection result in the third row is “Pterygium to be observed” category, the WP measured by the system is 3.25 mm, and the predicted result of the system is “Pterygium requiring surgery” category.

**Table 7:** Some samples whose prediction results are inconsistent with the doctor's visual inspection results.

**Origin**	**Cornea**	**Pterygium**	**Final**	**Visual inspection results**	**Prediction result**	**WP (mm)**
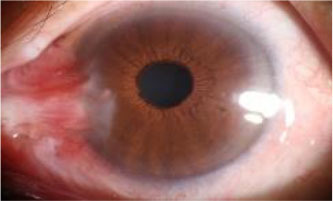	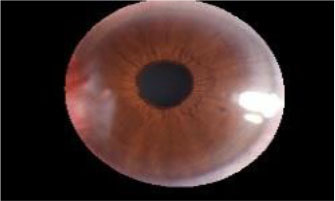	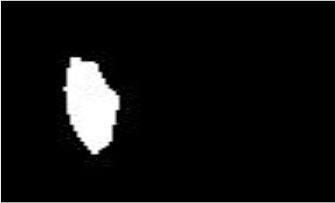	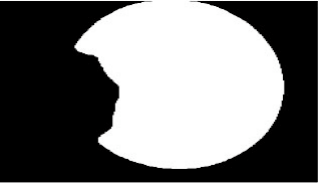	Surgery	Observed	2.56
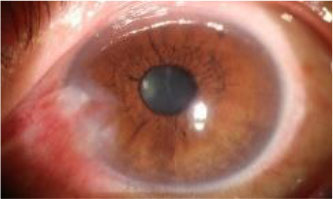	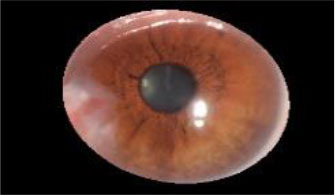	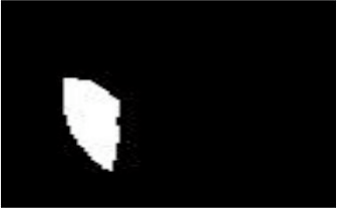	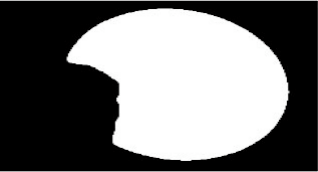	Surgery	Observed	2.67
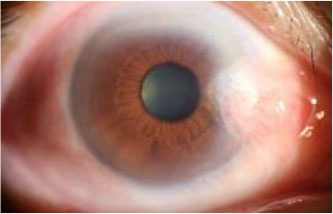	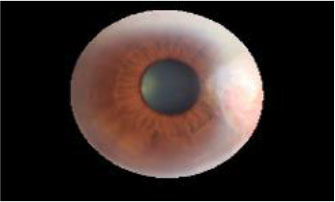	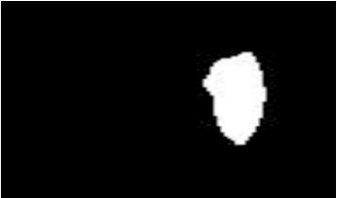	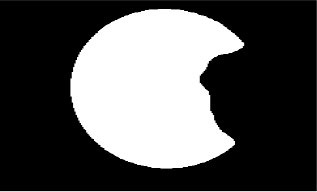	Observed	Surgery	3.25

**Figure 12:**
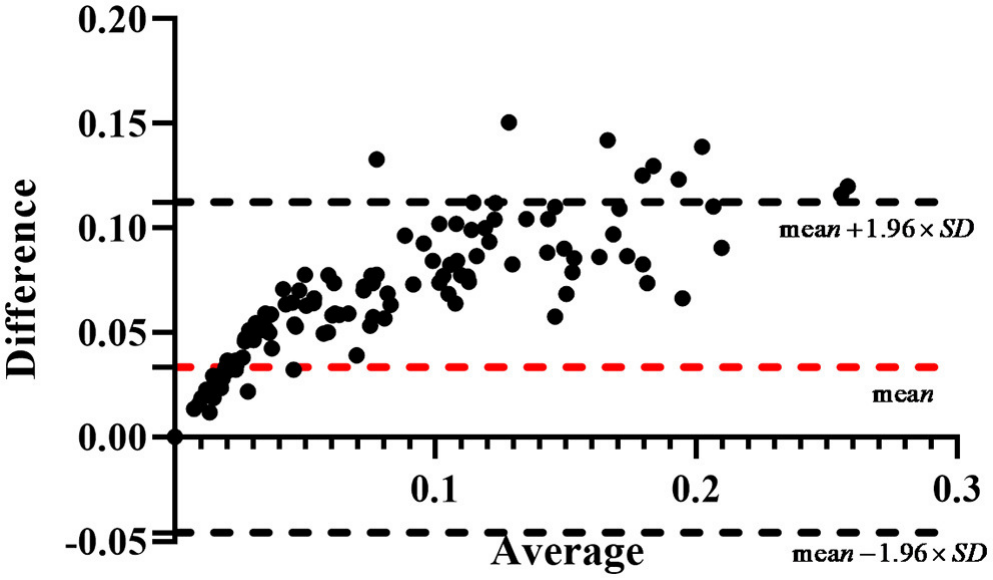
The result of the Bland-Altman consistency analysis.

In the measurement module, the calculated area of pterygium is obtained by estimating the actual area of cornea and multiplying the ratio of AP to AC. However, due to the lack of patients' actual area of cornea, it is difficult to obtain the actual area of pterygium for verification. Considering that in previous studies, some scholars have represented the area of pterygium by the ratio of AP to AC (30), we calculate the ratio of manually labeled AP to manually labeled AC as the ground truth to verify the accuracy of the measured results. Bland-Altman consistency analysis is carried out between the system's output ratio of AP to AC and the ground truth. The purpose of Bland-Altman consistency test is to compare whether the results obtained by different methods are consistent. By taking the average value of the two measurement data as the horizontal axis and the difference (area ratio of ground truth minus area ratio of measurement results) as the vertical axis, the result diagram of Bland-Altman consistency test is obtained. The consistency test result of the area ratio obtained by the two measurement methods in this study is shown in Figure 12. The red dotted line in Figure 12 is the mean value of the difference, and the two black dotted lines are the 95% consistency interval of the mean value of the difference. It can be seen that all the area ratio difference of test on 239 samples is >0, because in the process of pterygium segmentation, the segmentation accuracy is <100%, the area of segmented pterygium is smaller than the area of pterygium label, resulting error with the ground truth. In statistics, if the error is within 95% consistency interval, it means that the error is acceptable. In Figure 12, almost most of the 239 samples are within the 95% consistency interval, and only 9 samples are outside the consistency interval, it means that the results of area measurement method proposed in this study has good consistency with the results of the manual measurement method, so it has practical application value.

## Conclusion

Pterygium is an ocular surface disease, characterized by the proliferation of fibrous blood vessels on the conjunctiva. When pterygium significantly invades the cornea or even the pupil, it limits the movement of the eye and impairs vision, which means that the patient would need surgery to remove it. Therefore, measuring the pathological progress of pterygium is very important for designing surgical scheme. Since anterior segment images are usually used to assist the diagnosis in clinic, this study proposes a pterygium pathological progress measuring system using deep learning methods. By inputting the anterior segment image of patient, we can obtain the width of the pterygium which invades the cornea, the area of pterygium in the cornea, and its state (normal eye, pterygium to be observed, pterygium requiring surgery). These are crucial for realizing an accurate medical diagnosis, and it can conveniently assist doctors in the timely detection of a patient's pterygium status and arranging surgery strategies. The dataset used in this study was collected from the ocular surface diseases center at the Affiliated Eye Hospital of Nanjing Medical University. Through the evaluation of the system on the dataset, the effectiveness of the system was determined. The system can also be deployed in grassroots units and remote areas where medical resources are scarce so as to realize remote medical diagnosis.

The limitations of this study are as follows: due to the lack of professional measurement tools, we did not compare the measurement results with the actual width of the pterygium that invades the cornea, which means the measurement results can only be converted into classification results, and to be verified with the doctor' s visual inspection results; the cornea is an ellipse with a curved surface, but due to the lack of means to obtain the angle of the curved surface in this study, we assumed that it is a horizontal circle to calculate its area, which means there is still some error between the obtained area and the actual area.

In future study, we will measure the actual width of pterygium that invades the cornea using professional measurement tools, and compare the measurement results obtained by the system with the actual value to further verify the effectiveness of the system. Since the cornea is an ellipse with a curved surface, we will further study the method of obtaining the angle of the cornea surface to obtain a more accurate area of the cornea, which means we can obtain a more accurate measurement results output by the system.

## Data Availability Statement

The original contributions presented in the study are included in the article/supplementary material, further inquiries can be directed to the corresponding author/s.

## Ethics Statement

The studies involving human participants were reviewed and approved by Ethics Committee of the Affiliated Eye Hospital of Nanjing Medical University. The patients/participants provided their written informed consent to participate in this study.

## Author Contributions

CWan and YS: acquired, analyzed, discussed the data, and drafted the manuscript. CWang, JJ, and WY: acquired the clinical information and revised the manuscript. All authors contributed to the article and approved the submitted version.

## Funding

This research was funded by the Chinese Postdoctoral Science Foundation (2019M661832); Jiangsu Planned Projects for Postdoctoral Research Funds (2019K266); Jiangsu Province Advantageous Subject Construction Project and Nanjing Enterprise Expert Team Project.

## Conflict of Interest

The authors declare that the research was conducted in the absence of any commercial or financial relationship that could be construed as a potential conflict of interest.

## Publisher's Note

All claims expressed in this article are solely those of the authors and do not necessarily represent those of their affiliated organizations, or those of the publisher, the editors and the reviewers. Any product that may be evaluated in this article, or claim that may be made by its manufacturer, is not guaranteed or endorsed by the publisher.
